# Adaptive planning based on single beam optimization in passive scattering carbon ion radiotherapy for patients with pancreatic cancer

**DOI:** 10.1186/s13014-021-01841-2

**Published:** 2021-06-19

**Authors:** Yang Li, Yoshiki Kubota, Masahiko Okamoto, Shintaro Shiba, Shohei Okazaki, Toshiaki Matsui, Mutsumi Tashiro, Takashi Nakano, Tatsuya Ohno

**Affiliations:** 1 Graduate School of Medicine, Gunma University, Maebashi, Japan; 2 Department of Radiation Oncology, Harbin Medical University Cancer Hospital, Harbin, China; 3 Gunma University Heavy Ion Medical Center, Maebashi, Japan

**Keywords:** Carbon-ion radiotherapy, Adaptive planning, Pancreatic cancer, Accumulated dose assessment, Robustness of treatment

## Abstract

**Background:**

Daily anatomical deviations may distort the dose distribution in carbon ion radiotherapy (CIRT), which may cause treatment failure. Therefore, this study aimed to perform re-planning to maintain the dose coverage in patients with pancreatic cancer with passive scattering CIRT.

**Methods:**

Eight patients with pancreatic cancer and 95 daily computed tomography (CT) sets were examined. Two types of adaptive plans based on new range compensators (RCs) (AP-1) and initial RCs (AP-2) were generated. In AP-2, each beam was optimized by manually adjusting the range shifter thickness and spread-out Bragg peak size to make dose reduction by < 3% of the original plan. Doses of the original plan with bone matching (BM) and tumor matching (TM) were examined for comparison. We calculated the accumulated dose using the contour and intensity-based deformable image registration algorithm. The dosimetric differences in respect to the original plan were compared between methods.

**Results:**

Using TM and BM, mean ± standard deviations of daily CTV V95 (%) difference from the original plan was − 5.1 ± 6.2 and − 8.8 ± 8.8, respectively, but 1.2 ± 3.4 in AP-1 and − 0.5 ± 2.1 in AP-2 (*P* < 0.001). AP-1 and AP-2 enabled to maintain a satisfactory accumulated dose in all patients. The dose difference was 1.2 ± 2.8, − 2,1 ± 1.7, − 7.1 ± 5.2, and − 16.5 ± 15.0 for AP-1, AP-2, TM, and BM, respectively. However, AP-2 caused a dose increase in the duodenum, especially in the left–right beam.

**Conclusions:**

The possible dose deterioration should be considered when performing the BM, even TM. Re-planning based on single beam optimization in passive scattering CIRT seems an effective and safe method of ensuring the treatment robustness in pancreatic cancer. Further study is necessary to spare healthy tissues, especially the duodenum.

## Introduction

Carbon ion radiotherapy (CIRT) has emerged as a viable treatment option for unresectable pancreatic cancer in the last decades [1–4]. A multi-institutional study recently reported promising clinical outcomes in the overall survival and local control, which are superior to those obtained with conventional chemoradiation therapy or chemotherapy alone [1]. These are mainly attributable to its ability to provide a concentrated dose distribution to the target and better biological effect induced by a higher linear energy transfer [5, 6]. However, the carbon ion beam is highly sensitive to anatomical changes during treatment, which may cause a prominent dose distortion especially in pancreatic cancer.

Several studies have found that large position variation of pancreatic tumor and random gastrointestinal (GI) tract deformation exit between treatment fractions [7–10], unlike lung and liver tumors where good tumor reproducibility via tumor matching (TM) could account for interfractional deviations ensuring a robust treatment in CIRT [11–14]. Anatomical changes like GI deformation (gas volume changes) along the beam path may cause dose degradation, indicating that a poor dose distribution may be obtained even with TM [15]. Houweling et al. reported that using TM, the dose coverage was reduced by 10% on average and 17% for bone matching (BM) with CIRT [9]. Another study used the diaphragm structural matching to reduce positioning errors; however, this method seems to be helpful to estimate the tumor position, limiting the expected effects on maintaining the dose distribution [16]. Therefore, the current image-guided RT repositioning is limited in providing a robust CIRT treatment. Some studies attempted to improve the dose distribution by modeling daily gastrointestinal organs variations [17] or optimizing the original plan (OP) (e.g., worst-case optimization) [18]. These efforts help maintain the robustness of the dose coverage; however, the single plan applied for the entire treatment course cannot fully account for the patient-specific inter-fractional organ deformation in pancreatic cancer [19]. The full-scale re-optimization based on up-to-date imaging data seems necessary.

Adaptive radiotherapy has been considered as an effective technology for the treatment of pancreatic cancer in photon RT [8, 19]. However, only few studies have been conducted on adaptive CIRT due to various limitations [20]. Moreover, the passive scattering CIRT has a bottleneck that a patient-specific range compensator (RC) is needed at pretreatment making the performance of adaptive CIRT challenging. In addition, due to the use of single field irradiation on each treatment day, the evaluation methods for adaptive planning in photon RT seem not applicable to CIRT. There has been no study to address this issue. Therefore, this study aimed to explore the feasibility and possible benefits of employing adaptive CIRT based on single beam optimization. We proposed two adaptive plan protocols based on passive scattering CIRT for eight patients with pancreatic cancer. Dose deviations in respect to the OP were evaluated in both daily and accumulated dose distributions, as well as those investigated with BM and TM were used for comparison.

## Methods

### Patient and CT imaging acquisition

We studied eight consecutive patients with pancreatic cancer who were treated with passive scattering CIRT at our facility between March 2018 and February 2019. Patient characteristics can be found in Table 1. To obtain a satisfactory dose distribution by multiple beams, planning CT imaging was performed at the end of expiration in both the supine and prone positions because the fixed beam line is used at our facility (Aquilion LB, Self-Propelled, Canon Medical Systems, Japan) [15]. On the treatment day, daily CT sets were acquired by a separate CT under the same settings and conditions as the planning CT in the treatment room. Thus, a total of 16 planning and 95 daily CT sets were obtained (one CT set was missing because of the equipment failure). We performed gated irradiation within 30% of gating level.

**Table 1:** Patient characteristics

Patient no.	Sex	Age (year)	Tumor position	Daily CT sets	Treatment position	Volume changes (ml)		
						Tumor	Duodenum	Stomach
1	F	50	Body	12	SP + PR	25.8 ± 1.6	59.1 ± 8.1	252.0 ± 51.1
2	M	76	Head	12	SP + PR	22.9 ± 1.8	53.0 ± 10.0	-
3	F	83	Head	12	SP + PR	34.1 ± 2.2	102.7 ± 11.4	273.7 ± 57.1
4	M	81	Body	12	SP + PR	29.6 ± 3.5	63.7 ± 10.7	254.0 ± 81.2
5	F	51	Head	12	SP	24.1 ± 2.8	57.3 ± 9.8	215.0 ± 76.1
6	F	61	Body	12	SP + PR	24.8 ± 2.1	55.9 ± 11.4	221.9 ± 54.2
7	F	78	Body	11	SP + PR	21.6 ± 1.5	45.2 ± 5.6	152.6 ± 12.5
8	M	74	Head	12	SP + PR	42.5 ± 3.0	79.9 ± 14.6	201.2 ± 61.7

Patient 2 underwent gastrectomy before CIRT

*M* male, *F* female, *Body* pancreatic body, *Head* pancreatic head, *SP* supine, *PR* prone

### Original treatment plan

The treatment planning was established using a XiO-N system that employs a pencil beam algorithm (Elekta Sweden, Mitsubishi Electric, Japan) [21]. Four broad beams (angles: 0° (anterior–posterior), 90° (left–right), 270° (right-left), and 180° (posterior-anterior) based on a passive scattering technique were applied (180° beam was not applied for patient 5). Only one beam was delivered per treatment day as follows: 0°, 90°, 270°, 0°, 90°, 270°, 0°, 90°, 270°, 180°, 180°, and 180°. Our facility uses Gy (RBE) as the unit of the clinical dose, which was calculated based on the physical dose and relative biological effectiveness (RBE) [5]. The prescription dose was 55.2 Gy (RBE) in 12 fractions. The gross tumor volume (GTV) was determined for the primary tumor using a contrast-enhanced CT acquired at pretreatment. To protect the GI organs, a planning organ at risk volume (PRV)-GI defined as (GI boundary + 2 mm)—GTV was applied at our facility. In the first nine fractions, the clinical target volume 1 (CTV1) was defined as the GTV plus a 5-mm margin, including locoregional lymph nodes and neural plexus regions minus the PRV-GI margin; CTV2 for the last three boost fractions was generated by adding a 5-mm margin to the GTV, excluding the PRV-GI margin. Planning target volume (PTV) was established by adding a 3-mm margin to the CTV, excluding the PRV-GI margin. Treatment plans were performed to deliver at least 95% of the prescribed dose to 95% of the CTV and GTV (V95 > 95%). The dose coverage was slightly compromised when the distance between PTV and organs at risk (OARs) were close or overlapping. The dose to the most exposed 2 cc (D2cc) of the duodenum and stomach should receive < 44 Gy (RBE). A volume of the duodenum and stomach receiving 30 Gy (RBE) (V_30 [Gy (RBE)]_) should be < 10 cc as possible.

### Adaptive plan

The contours were delineated on each daily CT sets by two experienced radiation oncologists. We proposed two simulation adaptive plans as AP-1 and AP-2. AP-1 was generated under an idealized condition of no RC limitation, i.e., a new RC was applied for each beam according to daily anatomical changes; AP-2 used the same RC applied in each OP beam. The same beam angle arrangement as the OP was applied in both adaptive plans. In AP-1, optimal spread-out Bragg peak (SOBP) size and range shifter were chosen in XiO-N calculation ensuring a conformal dose at both proximal and distal edges. In AP-2, both SOBP and the range shifter were manually adjusted to satisfy the criteria ensuring the target dose reduction within 3% of the OP beam. Normally, it is necessary to reduce the OAR dose while maintaining the target dose as much as possible; however, it is difficult to generate a plan that meets these requirements in pancreatic cancer. In our proposed method, the priority strategy was to ensure target coverage. Multi-leaf collimator was optimized for both adaptive plans.

### Dose accumulation and evaluation parameters

To examine the dosimetric difference between the conventional positioning and adaptive plans, dose distributions with BM and TM were calculated. First, daily CT images were transferred to XiO-N for dose calculation (with the same parameters as the OP) after a rigid registration with the corresponding planning CT images with BM and TM using MIM Maestro (MIM Software, Cleveland, OH, USA). Then, dose distributions were warped and transferred to planning CT images in supine position using the DIR matrix (for one missing CT set, images acquired on the most adjacent treatment day were substituted). To reduce the DIR uncertainties, contour and intensity-based DIR (hybrid-DIR) was used [22, 23]. Lastly, accumulated doses were calculated with addition of twelve dose distributions. The accumulated doses for adaptive plans were obtained with the same process above after re-planning on each daily CT image in XiO-N.

The volume percentage receiving 95%, 90% of prescribed dose (V95, V90), the dose covered by 98% of volume (D98), and the mean dose were used to evaluate dose distributions for CTV and GTV. D2cc, mean dose, V_10 [Gy (RBE)]_, V_20 [Gy (RBE)]_, and V_30 [Gy (RBE)]_ were evaluated for the duodenum and stomach. The dosimetric differences between proposed methods and OP were examined. Moreover, the dose reduction of > 5% in CTV V95 was defined as unacceptable. The significance of statistical difference between methods was analyzed using the Wilcoxson signed-rank test and the Bonferoni correction was used to adjust the *P* value. The Friedman test was used for multiple comparisons. *P* < 0.05 was considered significant.

## Results

### Dosimetric difference in daily dose

**Fig. 1:**
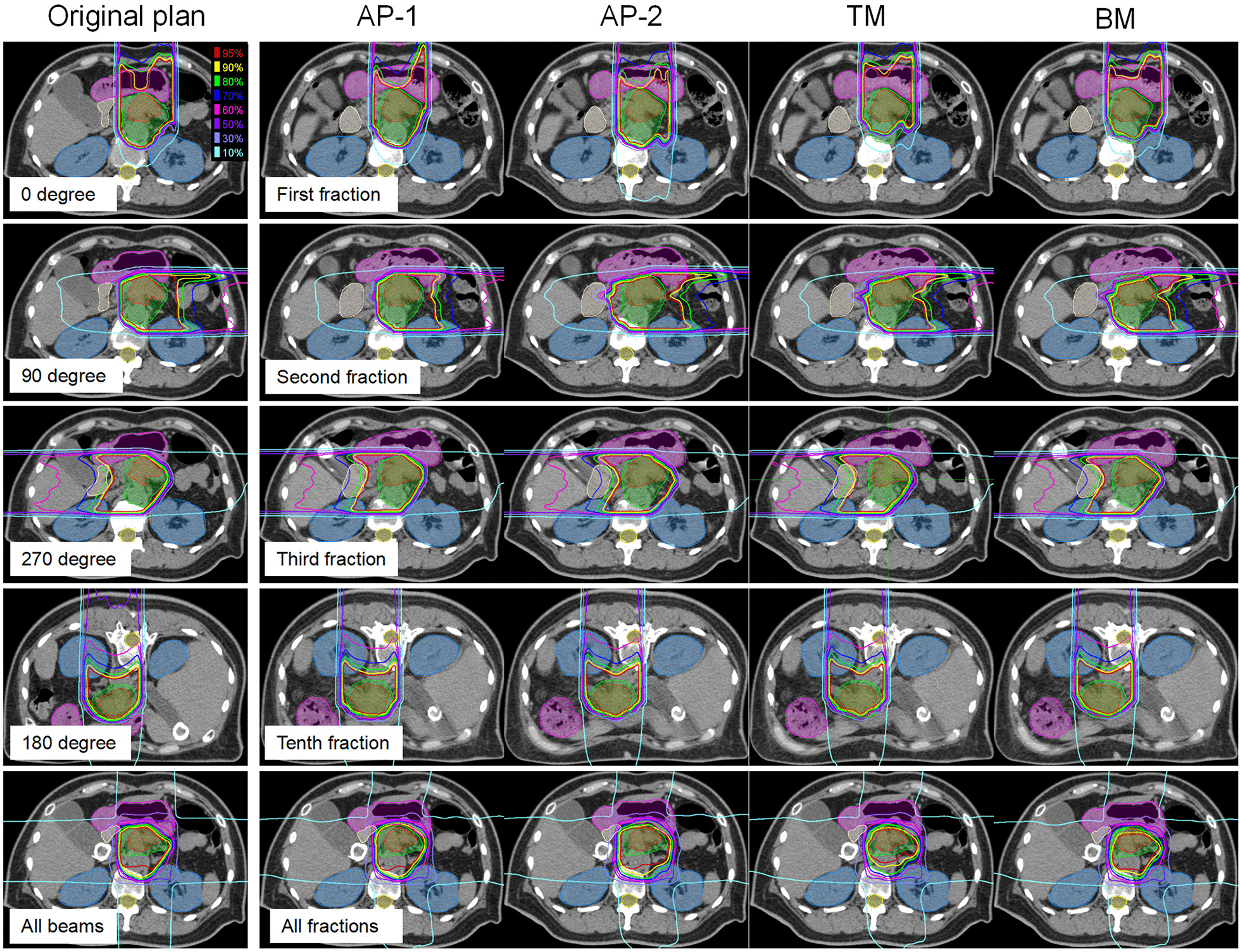
Example of dose distributions using AP-1, AP-2, TM, and BM (patient 1). The left panel shows dose distributions of the original plan at 0°, 90°, 270°, 180° beam angle, and all beams. The right panels from the top to the bottom show dose distributions with AP-1, AP-2, TM, and BM in the first, second, third, tenth, and all fractions, respectively. TM, tumor matching; BM, bone matching. Filled red, gross tumor volume; filled green, clinical target volume; filled blue, kidney; filled purple, stomach; filled white, duodenum; filled yellow, spinal cord

**Fig. 2:**
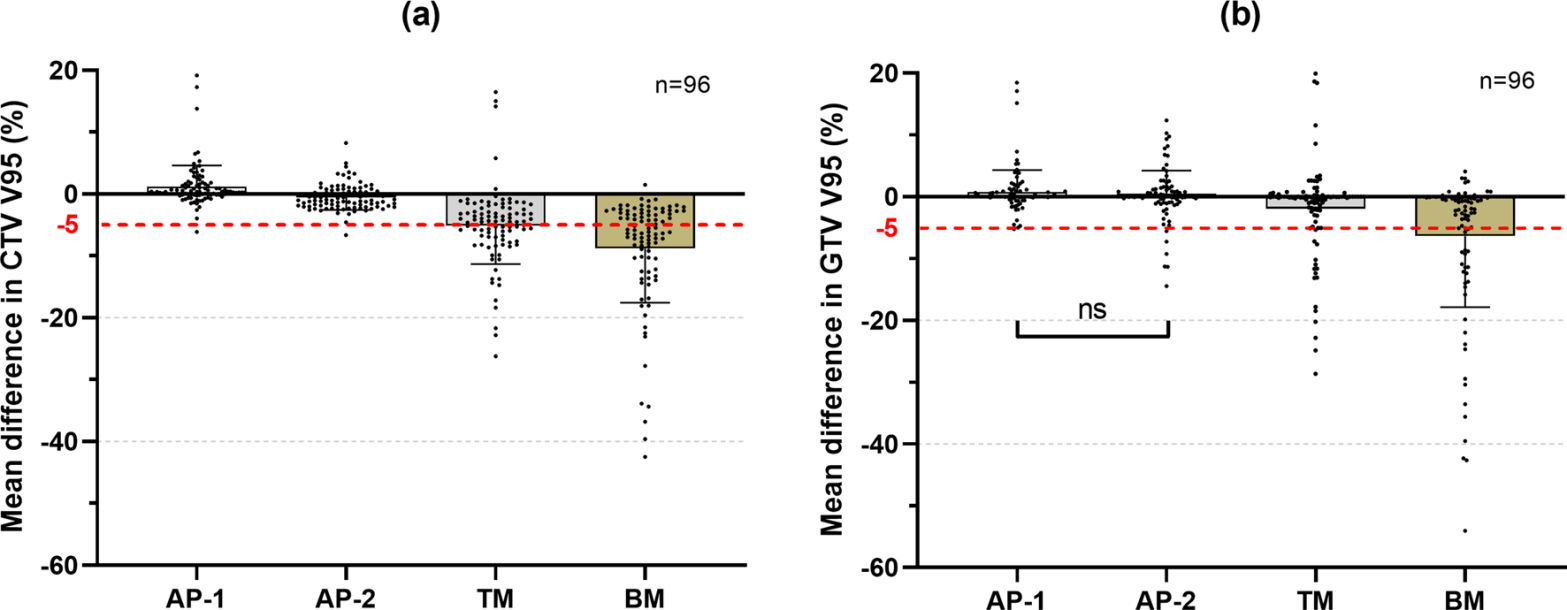
The mean dose difference of all fractions in CTV V95 (**a**) and GTV V95 (**b**) with AP-1, AP-2, TM, and BM. These differences are in respect to the original plan. GTV, gross tumor volume; CTV, clinical target volume; ns, *P* > 0.05. A significant difference was obtained between methods without ns (*P* < 0.05)

**Fig. 3:**
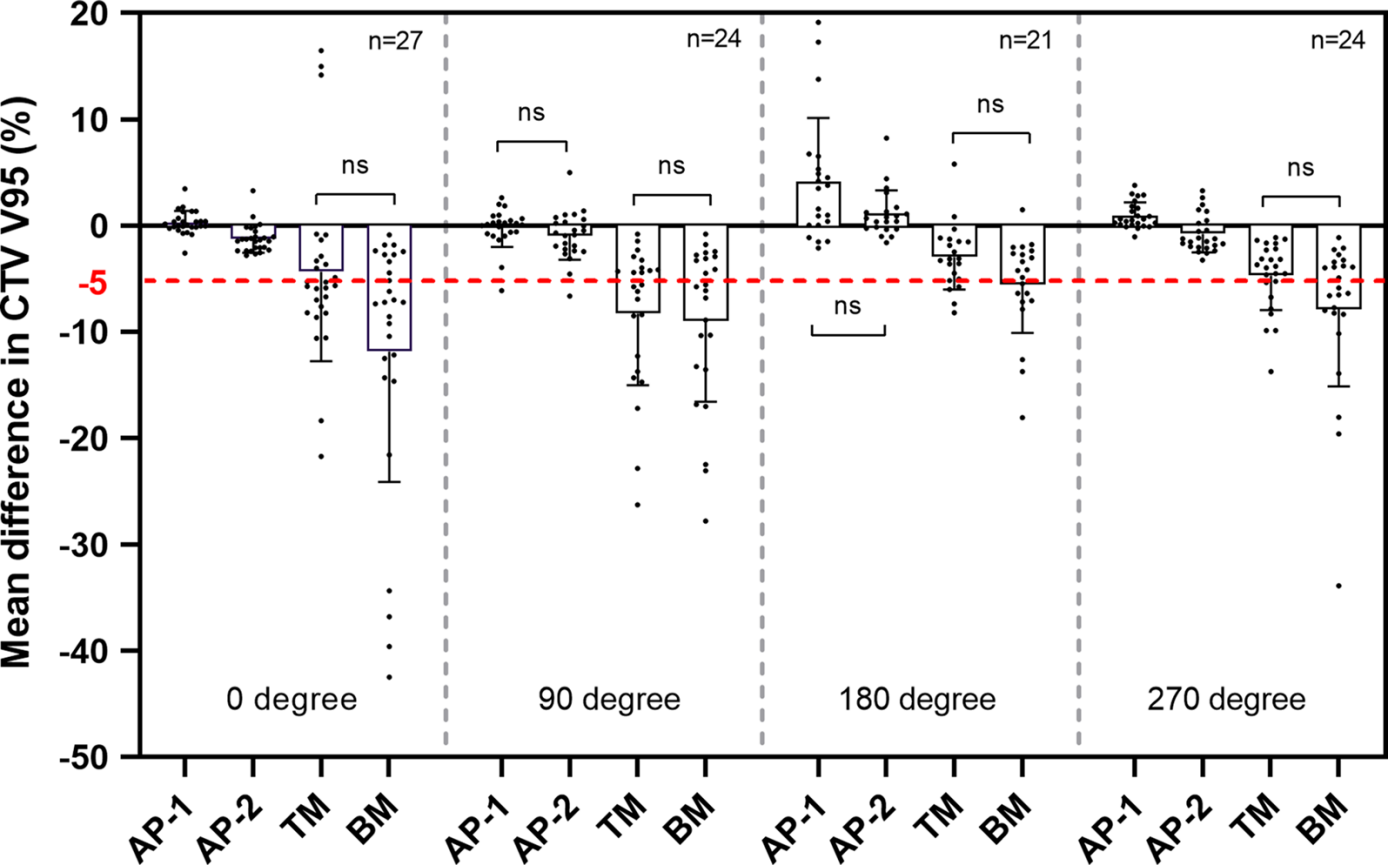
The mean dose difference of all fractions in CTV V95 at each beam angle. CTV1 doses were based on the first nine fractions. These differences are in respect to the original plan. CTV, clinical target volume; ns, *P* > 0.05. A significant difference was obtained between methods without ns (*P* < 0.05)

An example for dose distributions in four beam angles with AP-1, AP-2, TM, and BM is shown in Fig. 1. Using BM and TM, 56.8% and 47.5% of 96 fractional dose reductions were unacceptable in CTV, respectively, in contrast with 1% in AP-1 and AP-2. A significant difference was observed between methods (*P* < 0.001) (Fig. 2a). The dosimetric difference of mean ± standard deviation in CTV V95 was 1.2 ± 3.4, − 0.5 ± 2.1, − 5.1 ± 6.2, and − 8.8 ± 8.8 for AP-1, AP-2, TM and BM, respectively. In AP-2, four fractional CTV doses (one in 270° beam, three in 90° beam) failed to be meet the dose constraint because of severe dose distortion caused by anatomical changes; two fractional doses (both in 90° beam) were decreased by > 3% in AP-1 due to an extreme narrow distance between tumor and OARs. Adaptive plans had the best performance in average of CTV V95 at 180° and worst at 90°, but superior to BM and TM at all beam angles (Fig. 3).

### Dosimetric difference in accumulated dose

**Fig. 4:**
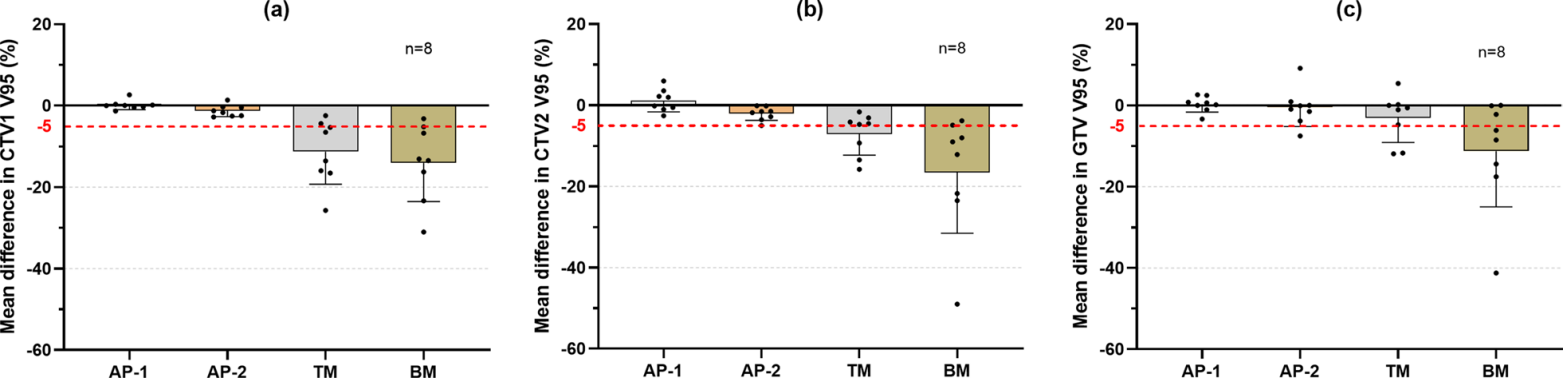
The mean dose difference of accumulated dose in CTV1 V95 (**a**), CTV2 V95 (**b**), and GTV V95 (**c**) with AP-1, AP-2, TM, and BM. CTV1 doses were based on the first nine fractions. These differences are in respect to the original plan. TM, tumor matching; BM, bone matching; GTV, gross tumor volume; CTV, clinical target volume

**Fig. 5:**
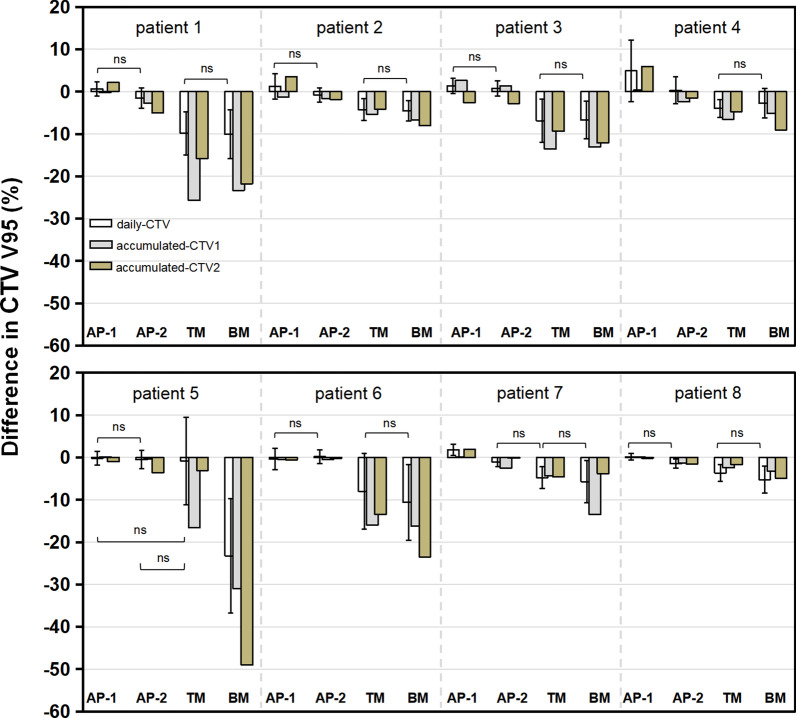
The dose difference of CTV V95 in each patient. CTV1 doses were based on the first nine fractions. These differences are in respect to the original plan. CTV, clinical target volume; ns, *P* > 0.05. A significant difference was obtained between methods without ns (*P* < 0.05)

For accumulated dose in CTV2 V95, the dose reduction of > 5% was observed in 3 and 6 patients with TM and BM, respectively; however, all patient doses were acceptable with AP-1 and AP-2 (Fig. 4). The dosimetric change in CTV2 V95 (%) was 1.2 ± 2.8, − 2.1 ± 1.7, − 7.1 ± 5.2, and − 16.5 ± 15.0 for AP-1, AP-2, TM, and BM, respectively. A better accumulated dose was obtained in CTV1 for adaptive plans. All dose reductions in CTV1 V95 (%) were < 3% in both AP-1 and AP-2. However, accumulated doses of 6 and 7 patients were unacceptable with TM and BM, respectively. The dosimetric difference in CTV1 V95 (%) was 0.1 ± 1.1, − 1.3 ± 1.4, − 11.3 ± 8.0, and − 14.0 ± 9.5 for AP-1, AP-2, TM, and BM, respectively. Figure 5 shows dosimetric differences in both daily and accumulated doses for each patient.

### Dosimetric difference in OARs

**Fig. 6:**
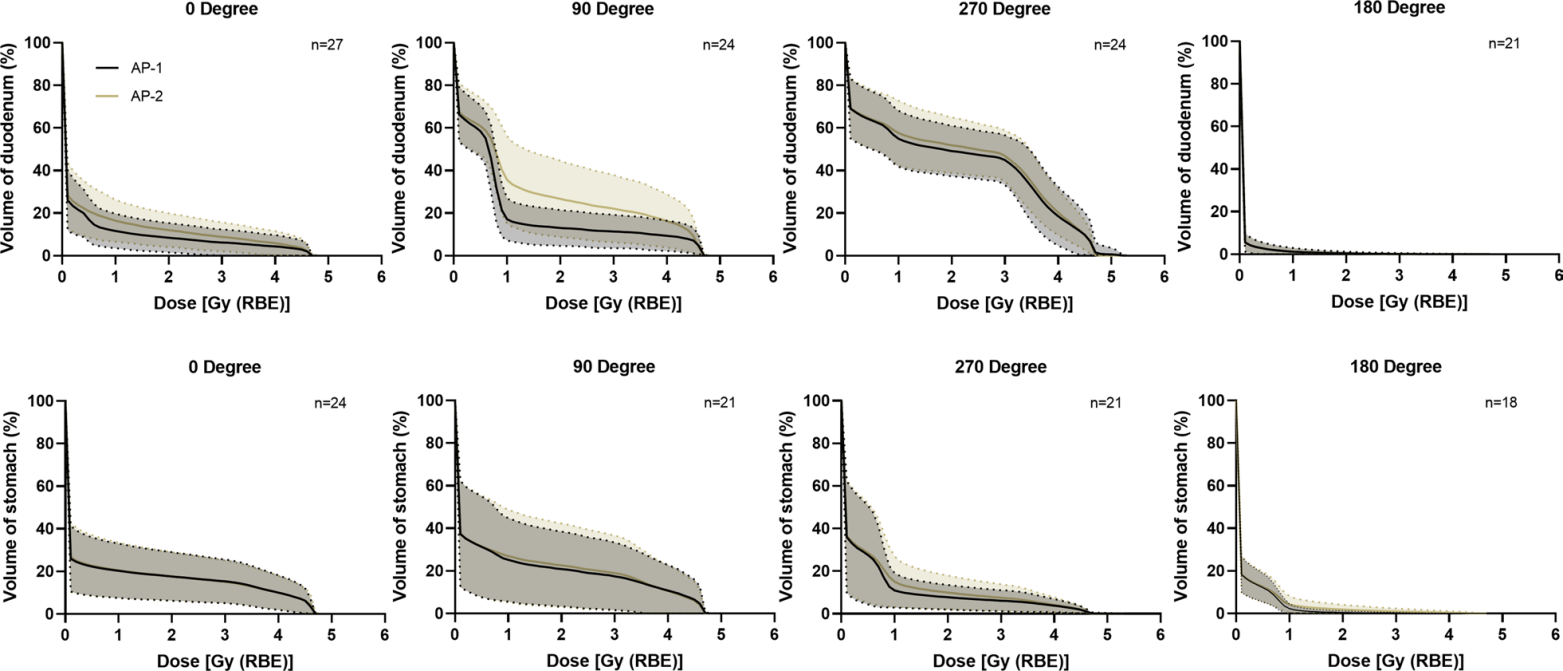
The average dose-volume histogram (solid line) of all fractions at each beam angle in the duodenum (upper) and stomach (below). Dashed lines show the standard deviation

Compared with the OP, the median duodenum doses in D2cc and mean doses were increased in all the methods, especially AP-2 (Table 2). In one case (patient 5), the dose exceeded the tolerance in D2cc when TM, BM, and AP-2 were applied. Lower median doses were obtained in V_10 [Gy (RBE)]_ but higher doses were observed in V_20 [Gy (RBE)]_ and V_30 [Gy (RBE)]_. The stomach doses in all the cases were within the tolerance in all the methods, and no significant differences were obtained as compared with the OP (*P* < 0.05). The average differences of the daily dose-volume histograms for the duodenum and stomach at each beam angle between the adaptive methods are shown in Fig. 6. Compared with AP-1, AP-2 caused a higher dose in the duodenum, especially at 90° beam angle; however, the delivered dose to the stomach was comparable at all angles between the two adaptive plans. The details of the dose-volume parameters are shown in Table 2.

**Table 2:** The dose-volume parameters of accumulated dose

Volume	Parameter	AP-1	AP-2	TM	BM	Original plan
CTV1	V95 (%)	99.4 (95.6–99.9)	97.5(94.3–99.4)	88.3 (79.4–97.5)*	84.8 (67.7–96.7)*	99.5 (93.0–99.9)
	V90 (%)	99.9 (99.1–100)	99.5 (98.7–100)	94.7 (89.1–99.7)*	91.2 (76.4–99.4)*	100 (97.8–100)
	D98 [Gy (RBE)]	40.1(38.4–40.8)	39.1 (37.9–40.3)	35.5 (32.1–39.1)*	33.0 (22.8–38.8)*	40.2 (37.1–40.7)
	Mean [Gy (RBE)]	41.4 (41.1–42.0)	41.3 (41.1–41.5)	40.9 (40.2–41.2)*	40.6 (38.5–41.1)*	41.4 (41.1–41.7)
CTV2	V95 (%)	96.7 (85.1–99.8)	91.8 (82.5–98.4)*	85.1 (81.0–98.3)*	80.5 (37.1–95.1)*	93.5 (86.1–100)
	V90 (%)	99.0 (91.1–100)	96.6 (88.5–99.8)	94.4 (89.9–99.6)	89.4 (46.5–98.3)*	98.5 (91.3–100)
	D98 [Gy (RBE)]	51.2 (43.8–54.5)	48.3 (44.4–52.8)	46.3 (43.4–52.8)	44.7 (21.6–50.0)*	49.3 (43.3–54.6)
	Mean [Gy (RBE)]	55.0 (54.3–55.4)	54.7 (54.0–55.2)*	54.4 (53.9–55.2)*	53.8 (44.6–54.9)*	54.8 (54.3–55.3)
GTV	V95 (%)	99.0 (88.2–100)	97.3 (90.7–100)	94.9 (85.8–100)	90.5 (44.4–100)*	98.5 (85.6–100)
	V90 (%)	99.8 (93.5–100)	99.6 (96.4–100)	99.0 (94.5–100)	96.7 (53.5–100)*	99.6 (90.9–100)
	D98 [Gy (RBE)]	53.6 (45.5–55.3)	52.1 (47.8–55.0)	50.8 (47.6–55.1)	48.0 (28.4–54.9)*	52.3 (43.3–55.1)
	Mean [Gy (RBE)]	55.2 (54.8–55.6)	55.1 (54.5–55.5)	55.0 (54.3–55.4)	54.6 (47.3–55.4)*	55.1 (54.3–55.5)
Duodenum	D2cc [Gy (RBE)]	28.5 (18.5–36.3)	32.1 (22.2–49.8)*	27.2 (20.8–47.4)	31.1 (18.2–46.9)	25.2 (21.3–40.2)
	V_10 [Gy (RBE)]_ (ml)	36.8 (21.8–79.5)	37.1 (22.5–82.5)	40.7 (24.3–79.0)	38.2 (27.7–81.0)	41.2 (22.5–82.7)
	V_20 [Gy (RBE)]_ (ml)	7.0 (1.7–25.6)	11.0 (3.1–52.2)*	5.6 (2.1–29.6)*	7.2 (0.9–25.5)*	3.5 (2.3–20.1)
	V_30 [Gy (RBE)]_ (ml)	1.4 (0.5–10.6)	2.9 (0.2–19.7)	1.9 (0.3–13.2)	2.5 (0.0–10.3)	1.0 (0.4–9.5)
	Mean [Gy (RBE)]	11.4 (6.8–15.1)	12.8 (7.8–22.4)	10.7 (7.8–20.5)	11.6 (7.8–19.2)	10.1 (7.7–15.6)
Stomach	D2cc [Gy (RBE)]	35.2 (19.0–40.1)	38.4 (23.7–42.3)	34.0 (18.9–38.4)	35.3 (21.1–44.0)	36.0 (16.9–43.6)
	V_10 [Gy (RBE)]_ (ml)	73.4 (23.3–106.1)	73.9 (26.1–106.3)	71.3 (17.2–107.6)	65.4 (19.2–100.1)	82.5 (20.9–113.8)
	V_20 [Gy (RBE)]_ (ml)	14.2 (1.7–45.2)	20.3 (3.8–48.9)	17.4 (1.6–49.7)	20.8 (2.4–45.4)	15.7 (1.1–51.2)
	V_30 [Gy (RBE)]_ (ml)	4.7 (0.2–18.0)	6.6 (0.6–21.6)	5.4 (0.1–22.3)	5.1 (0.3–20.7)	6.3 (0.1–26.9)
	Mean [Gy (RBE)]	7.6 (3.5–16.9)	7.6 (3.9–17.8)	7.3 (2.8–17.7)	6.8 (3.2–16.6)	8.9 (2.9–18.9)

Data are presented as median (range)

*TM* tumor matching, *BM* bone matching, *CTV* clinical target volume, *GTV* gross tumor volume, *V95 and V90* the percentage of the volume receiving 95% and 90% of prescribed dose, *D98* the dose coverage 98% of the volume. *V*_*n*_ the absolute volume receiving *n* Gy (RBE)

*P < 0.05 compared with original plan

## Discussion

In the present study, even performing TM, the dose distribution was decreased by 7% with respect to the OP, which was consistent with that of the previous studies [9, 15]. Although performing adaptive CIRT with a new RC for each fraction based on passive irradiation technology is impractical, AP-1 was proposed to examine the distance between doses with ideal condition and actual conditions. Only AP-1 allowed for a dose increase in average fractional dose changes in respect to the OP. AP-2 had a slight reduction, but prominently superior to BM and TM, in which approximately half of fractional dose reductions were > 5% in CTV V95 (Fig. 2). This finding indicates that adaption planning might be an effective strategy to improve the target dose, especially when employing the scanning technology where the doses would be similar to those with AP-1. However, in view of the actual treatment condition, AP-2 will be discussed in particular below.

Moriya et al. verified the effectiveness of the adaptive plan with range optimization method for three patients with lung cancer and one with abdominal lymph node metastases receiving passive scattering proton [24, 25]; one highlight of their study was that the adaptive plans were calculated using a developed range-optimization system, which can reduce labor burden. However, approximately 30 min are required for optimization calculation (for six beams), which seems still too long for online re-planning. In addition, only performing range optimization seems not enough in cases with large anatomical changes, e.g., the SOBP size proposed in OP may be too large or small to account for these changes. Therefore, full-scale re-optimization is crucial. Moreover, the same dose constraint as that of OP was used in their study to evaluate the adaptive plan. This strategy is similar to that used in photon RT, where doses based on all beams can be evaluated using the rotating gantry application [8, 26]. This is a conservative method that ensures a safe dose in OARs assuming that dose distribution in each fraction is similar. However, in single beam optimization, this strategy may not always work because the positional relationship between the target and OAR changes largely, especially in case of pancreatic cancer. Hence, we developed a method using the dose parameters of each beam in OP as a reference, and a dose reduction of ≤ 3% was used as the dose constraint for the target. Although XiO-N cannot automatically select the parameters that satisfy the dose constraint because it is not an inverse planning system, the algorithm to realize this system is simple. In fact, we manually performed the aforementioned optimization in only 10–15 min.

Interestingly, although only the target dose constraint was prioritized in this study, our strategy seems effective to balance the dose distribution between the target and OARs. The accumulated dose reductions in all patients were < 5% in CTV2 and < 3% in CTV1 in both adaptive plans; only one duodenal dose was clinically unacceptable in AP-2 (Fig. 4 and Table 2). Compared with BM and TM, re-planning proposal in this study maintained superior target dose coverage while ensuring a safe OAR dose in most patients (*P* < 0.05) (Table 2). One thing worth mentioning is that the extent of dose changes in CTV2 and CTV1 may be different. For example, in patient 5, an acceptable dose reduction (− 3.0%) in CTV2 was obtained with TM; however, a prominent dose degradation in CTV1 was observed (− 16.6%) (Fig. 5). Therefore, just evaluating the CTV1 or CTV2 dose was inadequate.

Comparing with AP-1 and OP, AP-2 had a comparable delivered dose in the stomach but a higher dose in the duodenum (Table 2). The fact is that the dose distortion caused by anatomical changes in BM and TM also existed in AP-2, especially in the 90° beam where large intestinal deformation usually occurred (Fig. 6). This is a limitation of AP-2. To address this problem, other beam angles such as posterior oblique direction seems a reasonable option. Beam angles from the 135° to 210° are considered to be effective to obtain a robust plan accounting for interfractional deviations by avoiding passing through the bowel gas [27–29]. However, considering the poor flexibility of the beam-angle arrangement (limited angles are available) in the fixed-beam port system, oblique beam selection remains challenging but merits close attention in the future study.

This study has some limitations. First, the contours were delineated manually, which is time-consuming and impractical for online adaptive RT. Developing a fast and accurate contouring system seems necessary, which will be considered in our future work. Second, this study was conducted in an idealized manner that daily adaption was performed to evaluate the accumulated dose. This is quite different from other adaptive protocols used in photon RT where re-planning is only performed for unacceptable cases examined prior to treatment [30]. However, considering the high ratio of unacceptable fractional dose reduction (approximately 50% in TM and BM in this study) in CIRT, daily re-planning seems necessary to ensure a safe treatment.

Third, the impact of intrafractional organ motion on dose distribution was not considered in this study [10]. The National Institute of Radiological Sciences (NIRS) has proposed a field-specific target volume (FTV) to account for the intrafractional variation in pancreatic cancer [27]. However, the FTV is just effective with the condition that the respiratory motion and organ deformation are reproducible during treatment, and possible interfraction deviations are not considered. Magnetic resonance (MR)-guided plan adaption seems a promising and ideal technology to consider both inter- and intrafractional deviations for pancreatic cancer [31–33], which is expected to be available in the particle therapy in a few years.

Another limitation is that a dose-constraint OAR value for each fraction is lacking, which may cause over irradiation to OARs when the whole treatment is completed. In this study, although dose constraints of OARs were assigned a lower priority than the target coverage, to minimize the OAR dose, such adaptive plan would be selected for AP-2: the one with an acceptable dose coverage in the target but minimal dose distribution in OARs. However, one patient’s duodenum dose was not tolerated in this study. Therefore, further studies considering both target and OARs is warranted to improve the adaptive CIRT. Lastly, DIR may cause uncertainties in dose accumulation. Kubota et al. [22] quantified the errors in hDIR and showed satisfactory dice similarity coefficients (DSCs) in CTV (0.94 ± 0.05); worse DSCs in the stomach and duodenum (0.85 ± 0.09 and 0.81 ± 0.06, respectively). These correspond to a 1.50% difference in median CTV V95 and 2.46% and 0.68% differences in median stomach and duodenum V50, respectively. Although, these errors seem within tolerance, a validation study with large samples is needed and improving the accuracy of DIR is necessary in further study.

## Conclusion

Improving the dose distribution for pancreatic cancer in CIRT with current image-guided positioning strategies is challenging. The possible dose reduction should be carefully considered when employing BM, even TM. The dose coverage in the target was significantly improved by re-planning in this study. Although AP-2 has uncertainties in OARs sparing due to RC limitation, the dose reduction constraint proposed for target seems effective to minimize OARs dose to some extent. However, considering a prominent dose increase in the 90° beam in the duodenum, other beam angle arrangements should be examined to improve this technology in the future. In fact, many issues still need to be addressed including the software and hardware aspects before performing adaptive CIRT in clinical practice, such as adjusting the range shifter and/or the ridge filter online is not yet possible at our facility, but it is not technically difficult. However, adaptive methods based on single beam optimization proposed in this study provide important evidence for the feasibility of implementing plan adaption with passive scattering CIRT in patients with pancreatic cancer.

## Data Availability

The datasets analyzed during the current study are available from the corresponding author on reasonable request.

## Abbreviations

CIRT: carbon ion radiotherapy
CT: computed tomography
RC: range compensator
TM: tumor matching
BM: bone matching
GI: gastrointestinal
OP: original plan
PTV: planning target volume
CTV: clinical target volume
GTV: gross tumor volume
RBE: relative biological effectiveness
PRV: planning organ at risk volume
SOBP: spread out Bragg peak
OARs: organs at risk
FTV: field-specific target volume
DSCs: satisfactory dice similarity coefficients
